# Modeling of inhomogeneous electromagnetic fields in the nervous system: a novel paradigm in understanding cell interactions, disease etiology and therapy

**DOI:** 10.1038/s41598-018-31054-9

**Published:** 2018-08-27

**Authors:** Jasmina Isakovic, Ian Dobbs-Dixon, Dipesh Chaudhury, Dinko Mitrecic

**Affiliations:** 1 Omnion Research International, Zagreb, Croatia; 2 Laboratory for Stem Cells, Department for Neurogenetics, Medical Genetics and Regenerative Neuroscience, Croatian Institute for Brain Research, School of Medicine, University of Zagreb, Zagreb, Croatia; 3 Division of Science, New York University Abu Dhabi, Abu Dhabi, United Arab Emirates

## Abstract

All major processes in the nervous system depend on interactions between cells and nerve fibers. In this work we present a novel model of inhomogeneous electromagnetic fields originating from nerve fibers and delineate their influence on cells. By expanding Hodgkin-Huxley’s applied current into axial current, governed by${J}_{i}^{j+1}=K\times {J}_{i}^{j}$, we reveal that cell-with-neuron interactions are regulated by the strength of the electromagnetic fields, which are homogeneous up to *2.066* *μm* or *6.606* *μm* away from neurilemma and axolemma, respectively. At the nodes of Ranvier, these fields reach strengths of 3.0 × 10^−12^*T*, while at the myelinated segments they only peak at 2.3 × 10^−12^*T*. These are the same fields which are, due to inhomogeneity, detected as 1,000 times weaker by magnetoencephalography. Considering the widespread occurrence of neurodegenerative disorders, our model reveals that a 50% demyelination increases the field strength by 0.35 × 10^−12^*T*, while a complete demyelination increases it by 0.7 × 10^−12^*T*. Since this suggests that the inhomogeneous electromagnetic fields around neurons play a role in physiological and pathological processes, including cell-to-neuron and cell-to-cell communication, their improved understanding opens up new therapeutic strategies based on electromagnetic field modulation or cell’s surface charge alteration.

## Introduction

Neuroinflammation is one of the central elements in majority of pathological processes in the nervous tissue. It includes attraction, migration and accumulation of cells which support cascade of events participating in both damage and regeneration of the tissue. One of the first steps, needed for cells to cross the blood brain barrier, is their attachment to endothelium over cell adhesion molecules (CAMs). By changing the conformation of their central elements - integrins, CAMs interact over cations (Mg, Na, Ca) which, in turn, over involvement of specific quantities of charges - either increase or decrease cell adhesion^1^. Interestingly, although neuronal cell bodies do not express CAMs, they are present throughout the cells of white matter, including the myelin sheath^2^. Indeed, there is a growing list of evidence that the cell membrane elements which are involved in regulation of cell charge do have numerous roles. Thus, it has been shown that Na/K-ATPase pump has a significant influence on cell motility and cell invasiveness^3^, cancer propagation^4^, apoptosis^5^ and scar formation^6^. In addition, a growing list of evidence is revealing that adhesion of immune cells, modulated over various mechanisms, does not only support, but can trigger immune reaction^7,8^. More precisely, it has been recently suggested that changes in the cell’s membrane charge may facilitate the recruitment of signaling molecules to the inner leaflet of the plasma membrane in cell receptors microclusters^9,10^. Similar observation about the role of the surface charge were also obtained by experiments in which the charge has been modulated by biomaterials, acting as “immunocamouflage”^11^. Taken altogether, it is timely to hypothesize that the cell’s surface charge has much more important roles in cell adhesion and activation of interactions, in both physiological and pathological processes, than previously thought.

In this work we model inhomogeneous electromagnetic (EM) fields around nerve fibers and their interaction with cells present in the nervous system. As a cell’s membrane is made up of a phospholipid bilayer with proteins being embedded in between, it carries a negative charge. Next to the phospholipid bilayer, the instantaneous relative negative charge on a cell is also due to the influx and outflux of chlorine ions through the cystic fibrosis transmembrane conductance regulator gates (CFTR) and contributions of the Na/K-ATPase pump. With this, the negative charge on the surface of a cell, net electricity, is the result of the trans-membrane potential, levels of charged components on the plasma membrane and the activities of ion channels on the plasma membrane.

Nervous tissue is composed of neuronal cell bodies, which are mostly grouped in the gray matter, and cell projections of various lengths which form nerve fibers. Myelinated fibers are cell projections surrounded by a myelin sheath that is made up of a lipid layer formed by Schwann cells or oligodendrocytes. During propagation of an action potential (AP), each of the myelinated segments and the nodes of Ranvier, periodic gaps in the myelin sheath, carry a negative external surface charge and a positive internal charge within the neuron. As the AP propagates, it causes an acquisition of net charge in the extracellular and depletion of charge in the intracellular space through intracellular and extracellular current flow. In this paper we describe how this current flow generates a difference in membrane potential across the membrane and axial current flow from previous to consecutive axonal segments, resulting in generation of an electromagnetic field around the neurons. As opposed to the uniform static magnetic field that is being measured by magnetoencephalography (MEG), here we model and precisely describe how temporal and spatial propagation of an action potential, and subsequent intracellular and extracellular current flow, generates an inhomogeneous time-varying electromagnetic field which serves as a source of the field recorded by MEG. Due to the insulating nature of the myelin sheath, this field presents itself as a magnetic field at myelinated regions of the axon and an electromagnetic field at the nodes of Ranvier.

By using advanced mathematic modeling, supported by experimental proof of concept, we demonstrate that the electromagnetic fields generated around the neurons significantly, in extent much higher than thought before, influence the migration, adhesion and activity of negatively charged cells. Due to the charged nature of all cells within the human body, our model provides a new mechanism that could be exploited to direct stem cells, T- and B-lymphocytes, natural killer cells (NKC) and other cell types onto the injury site, or prevent their further activation. This also introduces a new set of parameters in our understanding of the onset and progression of neuroinflammatory events and opens new directions for potential therapeutic targets, both in the form of cell surface charge depletion and the reversal of electromagnetic field direction around axons.

## Results

**Figure 1:**
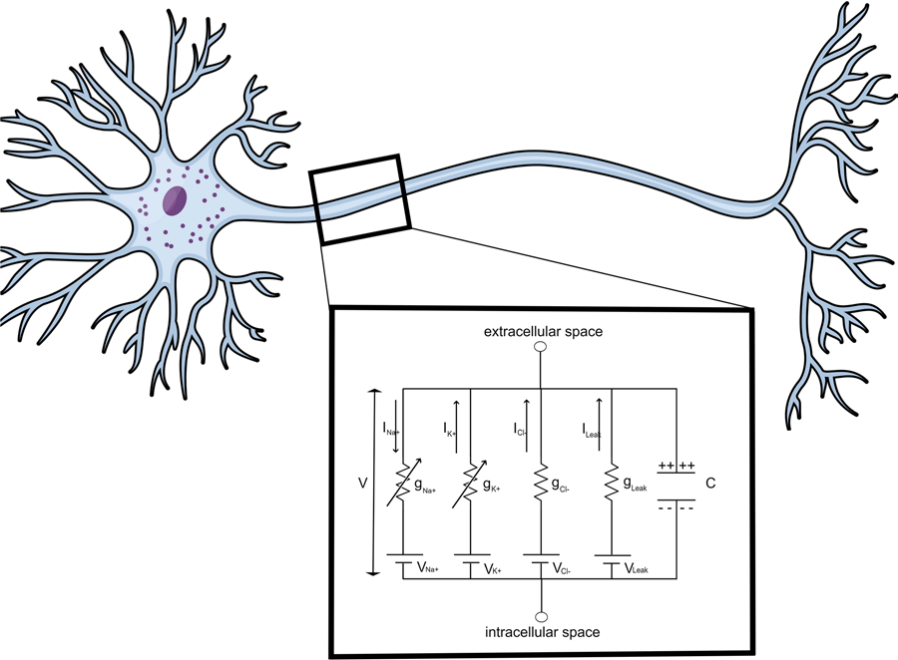
Hodgkin-Huxley Model circuit representation of the neuronal membrane. The circuit consists of a capacitor cell generating capacitive current and ion flow across the cell membrane, generating the ionic current. Ion channels and ionic currents cause an acquisition of net charge in the extracellular and depletion of charge in the intracellular space. This leads to a potential difference, V, across the neuronal membrane. (Source: Mind the Graph on https://mindthegraph.com/, used under the CC BY-SA license (https://creativecommons.org/licenses/by-sa/4.0/deed.en)/derivative of the original).

Starting from the Hodgkin-Huxley’s circuit representation of an axon (Fig. 1), this paper transforms and significantly upgrades their model in a novel way by including spatial and temporal progression of an action potential and the corresponding changes to ionic and capacitive current flow through modifying the applied current term to include axial, intracellular longitudinal current propagation. Moreover, we propose the existence of a time-varying inhomogeneous electromagnetic field around axons which results from the variability in the current flow and can significantly impact cell motion within the central nervous system (CNS).

To begin, for a cell to interact with any electromagnetic field, such as the time-varying EM fields around axons this paper puts forth, it has to be a charged particle in relative proximity to the source of the field. This has been elaborated by Kirschivink *et al*.‘s who showed that, throughout the CNS, white blood cells behave like “diamagnetic microparticles in plasma”^12^. The idea that the cells themselves carry a magnetic property implies that they, themselves, are susceptible to be influenced by electromagnetic forces generated by the EM fields around neurons.

**Figure 2:**
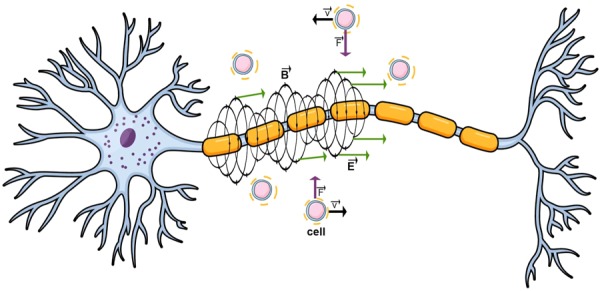
Forces acting upon cells in vicinity of the neuron. Once the cells penetrate the blood brain barrier and come in vicinity of the electromagnetic fields generated around neurons, their migration and adhesion begin to be influenced by the electric and magnetic forces exerted on them. Whilst the magnetic field acts circularly around the neuron, the electric field spreads perpendicularly to it. Both of these fields exert forces on cells which depend on the value of the external charge on the cells as well as their incident angle and could, therefore, either deflect or attract the cells. (Source: Mind the Graph on https://mindthegraph.com/, used under the CC BY-SA license (https://creativecommons.org/licenses/by-sa/4.0/deed.en)/derivative of the original).

Combining the notion that an axon can be modeled as a uniform conducting cable with neural impulses speeding through it, as in the Hodgkin-Huxley’s Model, this paper explains how a time-varying inhomogeneous electromagnetic field is generated around it and quantifies its strength and distances it can act upon. This EM field, in turn, exerts electric and magnetic forces on the cells within the CNS, potentially influencing their migration and adhesion in pathological conditions where the cells have already penetrated the blood brain barrier (Fig. 2).

Even though the axon surrounded by a dielectric myelin sheath is electrically insulated, and the polarization that the electric field inside the neuron causes almost gets dissipated by the exchange of potassium and sodium within the axon, a magnetic flux passes right through the myelin sheath resulting in an occurrence of an electromagnetic fields at the nodes of Ranvier and pure magnetic fields at the myelinated regions.

### Modeling the spatio-temporal propagation of the action potential through longitudinal and axial current flow

According to its current distribution, the cell membrane can be represented as a parallel circuit. The total current flowing through the circuit can then be described as the sum of all the ionic currents and those currents due to non-specific ionic leaks. Since potassium, sodium and chloride ion channels play the most significant role in the charge distribution and flow both in intracellular and extracellular space, those are the channels included in this model. By the Hodgkin-Huxley Model, the chloride and other ion channels are included in the leak current and conductance distribution^13^.

To account for the change in membrane capacitive and ionic potential along the neuron, the Hodgkin-Huxley Model was transformed in a compartmental method manner for spatio-temporal propagation of the action potential. This was done by using ordinary differential equations (ODEs) and adding additional coupling terms to relate the spatial and temporal dependence between different neuronal compartments to yield

$${C}_{m}\frac{{V}_{i}^{j+1}-{V}_{i}^{j}}{{\rm{\Delta }}t}=\frac{1}{2}(\frac{d}{4{R}_{i}}\frac{{V}_{i+1}^{j+1}-2{V}_{i}^{j+1}+{V}_{i-1}^{j+1}}{{\rm{\Delta }}{x}^{2}}-\,{G}_{m}{V}_{i}^{j+1}-\,{J}_{i}^{j+1}+\frac{d}{4{R}_{i}}\frac{{V}_{i+1}^{j}-2{V}_{i}^{j}+{V}_{i-1}^{j}}{{\rm{\Delta }}{x}^{2}}-{G}_{m}{V}_{i}^{j}-{J}_{i}^{j})$$

Here, i is the spatial and j the temporal index, *R*_*i*_ is the resistance, *d* is the diameter, *G*_*m*_ is the membrane conductance. All the variables are denoted per unit length of an axon.

Since ${J}_{i}^{j}$ is seen as the external current applied to the initial axonal segment, in all current models ${J}_{i}^{j+1}$ is set to 0. As the model has, thus far, been simplified not to include the current applied at the following segment, our model aims to shed light on the existence of longitudinal and axial currents throughout the axon which serve as initiators of impulse propagation at the following segment.

Even though Hodgkin-Huxley accounted for ionic and capacitive current flow during an action potential, they failed to account for the temporal and spatial dependency of the currents. With this in mind, here we developed a model that, for the first time, takes into account the depolarization cycle of the cell membrane which produces the action potential and can last *1-2 ms* and reoccur ≈100× per second. By adding additional terms in the form of axial, *I*_*ax*_, and longitudinal currents, *I*_*lng*_, we replaced the applied current term in the Hodgkin-Huxley Model and expanded their theory. As the axial current results from the spatio-temporal propagation of an action potential, it is defined as

$${I}_{ax}={I}_{i}^{j+1}$$

**Figure 3:**
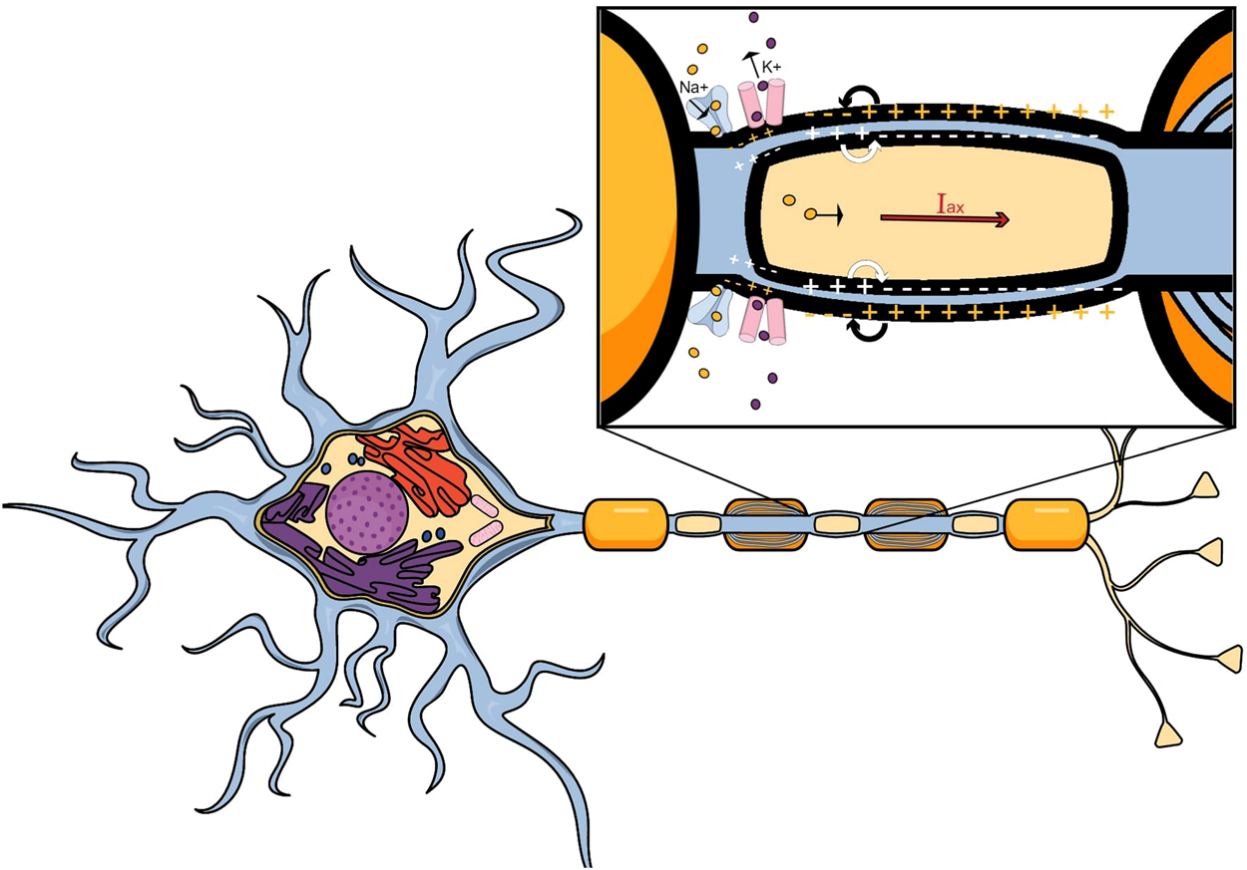
Proposed axial and longitudinal current flow generation within a neuron. Different from current models which approximated the electric current flow through the neuron as a continual flow of ions through sodium, potassium and leak channels which is constant in time during an action potential, we propose an additional term of applied current to be added to each consecutive axonal segment. This axial current is a product of the continuous ion flow through the cell’s membrane and its transversal components and, with that, aids action potential propagation and attenuation. (Source: Mind the Graph on https://mindthegraph.com/, used under the CC BY-SA license (https://creativecommons.org/licenses/by-sa/4.0/deed.en)/derivative of the original).

Taking into account the constant flow of ions through the membrane, it is expected that some component of the current flow will be in longitudinal direction, parallel to the membrane itself and would, with that, serve as the applied current to the following axonal segment - initiating further action potential propagation (Fig. 3). To account for the axial current, the Hodgkin-Huxley model was solved using the Crank-Nicholson Method to include temporal and spatial propagation of an impulse along the neuron; resulting from the ionic and axial current flow contributions.

Once an action potential starts propagating, it spreads as a cycle of depolarization and hyperpolarization along the neuronal membrane as an ionic flow through sodium, potassium and other leak channels. But, since the axial current generated by the passive spread of an action potential aids in initiating the spread of the impulses in the following neuronal segment, it should be included in the Crank-Nicholson Method for solving the Hodgkin-Huxley cable equation. This then suggests that the applied current density at the following neuronal segment is a function of the original applied current, in our case the repurposed axial current, so we established the following relation

$${J}_{i}^{j+1}=K\times {J}_{i}^{j}$$

Here K is a constant, a quantitative evaluation of what fraction of the original applied current is being transfered to the following axonal segment in the form of an axial current, codependent on the workings of the ion channels in the following segment. To obtain a measure of *K*, a clear relationship between ${J}_{i}^{j+1}$ and ${J}_{i}^{j}$ needed to be set.

Since an action potential propagates along a neuron for great distances, if ${J}_{i}^{j} > {J}_{i}^{j+1}$ the signal would dissipate as a function of ${J}_{i}^{j}$ which, physiologically, is an impossibility. This then suggests that axial current contribution to the following segment and, with that, the constant K, should attenuate the current density in the following segment or maintain an average value along the neural network in order not to dissipate the signal towards the ends of the action potential path to enable uniform propagation.

With this, we propose that

1 $$K > 0,{\rm{such}}\,{\rm{that}}\,{J}_{i}^{j} < {J}_{i}^{j+1}$$

and should enable a uniform and attenuated action potential propagation. This relation suggests that, in order to maintain uniform, or attenuate, AP propagation, on top of ionic current flow and depolarization and hyperpolarization cycles of the neural membrane, longitudinal and axial currents have to spread along the axon with the AP impulse.

Now that the relationship between current and current density propagation amongst consequent axonal segments has been defined, the values of current density at the start and at the following time step, *j* + 1, had to be found. Since $\overrightarrow{J}$ is the measure of density of the electric current, it is defined as

$$\overrightarrow{J}=\frac{\overrightarrow{I}}{A}$$

and the axial current, $\overrightarrow{I}$, generated by ionic and longitudinal current flow is defined by

$$\overrightarrow{I}=\gamma {g}_{K}(V-{V}_{K})+\delta {g}_{Na}(V-{V}_{Na})+\eta {g}_{L}(V-{V}_{L})+{I}_{lng}$$

As *g*_*K*_, *g*_*Na*_ and *g*_*L*_ are, in their ODE form, functions of time and then the longitudinal current, *I*_*lng*_, as well as the overall neuronal current have to be functions of time and space as well. The longitudinal current is set to be the current that propagates along the length of the neuron due to the potential difference across the membrane and contributes to the overall axial current value and direction.

In order to account for the axial current being a function of the longitudinal current as well as various contributions from the ionic flow, constants *γ*, *δ* and *η* are added to signify the components of the ionic current that are conducive to axial current propagation. As only a fraction of the ionic current flow will be giving rise to axial current flow, the constants have to follow the relation

$$\gamma {g}_{K}(V-{V}_{K}) < {g}_{K}(V-{V}_{K})$$

$$\delta {g}_{Na}(V-{V}_{Na}) < {g}_{Na}(V-{V}_{Na})$$

$$\eta {g}_{L}(V-{V}_{L}) < {g}_{L}(V-{V}_{L})$$

This means that

$$0 < \gamma ,\delta ,\eta  < 1$$

*γ*, *δ* and *η* are constants greater than 0, as there can’t be a negative contribution towards the axial current flow. Any negative contribution towards the current flow would signify dampening of AP propagation, as opposed to its attenuation.

Next, evaluating the ionic channel contributions to the passive and active spread of the action potential, the contribution of other, leak channels could be dispelled as the values of sodium and potassium ion channel conductance and contribution overpower the leak channels. This then helped us cancel out the term including *g*_*L*_ which then means that, solving for the spatio-temporal indexes using the Crank Nicholson Method, axial current components can be defined as

$$\begin{array}{rcl}{I}_{i}^{j} & = & \gamma {\bar{g}}_{K}{[n(j-1)+{\rm{\Delta }}x({\alpha }_{n}(j-1)(1-n(j-1))-{\beta }_{n}(j-1)n(j-1))]}^{4}\\  &  & \times ((V(j-1)+{\rm{\Delta }}x\frac{{I}_{ion}}{C})-{V}_{K})+\delta {\bar{g}}_{Na}[m(j-1)\\  &  & +{\rm{\Delta }}x({\alpha }_{m}(j-1)(1-m(j-1))-{\beta }_{m}(j-1)m(j-1)){]}^{3}\\  &  & \times [h(j-1)+{\rm{\Delta }}x({\alpha }_{h}(j-1)(1-h(j-1))\\  &  & -{\beta }_{h}(j-1)h(j-1))]((V(j-1)+{\rm{\Delta }}x\frac{{I}_{ion}}{C})-{V}_{Na})+R[V(j-1)+{\rm{\Delta }}x\frac{{I}_{ion}}{C}]\end{array}$$

$$\begin{array}{rcl}{I}_{i}^{j+1} & = & \gamma {\bar{g}}_{K}{[n(j)+{\rm{\Delta }}x({\alpha }_{n}(j)(1-n(j))-{\beta }_{n}(j)n(j))]}^{4}\\  &  & \times ((V(j)+{\rm{\Delta }}x\frac{{I}_{ion}}{C})-{V}_{K})+\delta {\bar{g}}_{Na}[m(j)+{\rm{\Delta }}x({\alpha }_{m}(j)(1-m(j))\\  &  & -{\beta }_{m}(j)m(j)){]}^{3}[h(j)+{\rm{\Delta }}x({\alpha }_{h}(j)(1-h(j))-{\beta }_{h}(j)h(j))]\\  &  & \times ((V(j)+{\rm{\Delta }}x\frac{{I}_{ion}}{C})-{V}_{Na})+R[V(j)+{\rm{\Delta }}x\frac{{I}_{ion}}{C}]\end{array}$$

From here, the current densities ${J}_{i}^{j}$ and ${J}_{i}^{j+1}$ are

$${J}_{i}^{j}=\frac{1}{A}{I}_{i}^{j}$$

$${J}_{i}^{j+1}=\frac{1}{A}{I}_{i}^{j+1}$$

where A and R have both been defined as area and resistance of the neuron.

Therefore, our new model, for the first time, includes the current applied at the following axonal segment, in the form of our proposed axial current, as well as a term quantifying the temporal propagation of current density within an axon which was, previously, excluded and neglected in such models.

### New model describes spatial propagation of the action potential and yields exact current, voltage and current densities at each axonal segment

Starting from the Crank-Nicholson method of solving the Hodgkin-Huxley model of an axon, this section of the paper defined a novel term ${J}_{i}^{j+1}$ as a current density at the following axonal segment that results from axial current propagation. From here, a system of coupled ordinary differential equations was obtained that describes the properties of a neuron as an excitable cell, through voltage-gated ion channels and axial current propagation along the neuron during the spread of an action potential. In this system, a novel method for both applied current density and propagating current density was derived to include axial current propagation, which should also contribute to the overall current and voltage distribution and spatio-temporal propagation of the action potential.

2 $$(\begin{array}{rcl}{C}_{m}\tfrac{{V}_{i}^{j+1}-{V}_{i}^{j}}{{\rm{\Delta }}t} & = & \frac{1}{2}(\tfrac{d}{4{R}_{i}}\tfrac{{V}_{i+1}^{j+1}-2{V}_{i}^{j+1}+{V}_{i-1}^{j+1}}{\Delta {x}^{2}}-{G}_{m}{V}_{i}^{j+1}\\  &  & -{J}_{i}^{j+1}+\tfrac{d}{4{R}_{i}}\tfrac{{V}_{i+1}^{j}-2{V}_{i}^{j}+{V}_{i-1}^{j}}{{\rm{\Delta }}{x}^{2}}-{G}_{m}{V}_{i}^{j}-{J}_{i}^{j}))\\ {J}_{i}^{j} & = & \frac{1}{A}{I}_{i}^{j}\\ {J}_{i}^{j+1} & = & \frac{1}{A}{I}_{i}^{j+1}\\ A & = & 2\pi rx+2\pi {(r)}^{2}\\ R & = & \frac{\rho x}{2\pi r(x+r)}\\ {I}_{i}^{j} & = & \gamma {\bar{g}}_{K}[{n}_{i}^{j-1}+{\rm{\Delta }}x({\alpha }_{n,i}^{j-1}\mathrm{(1}-{n}_{i}^{j-1})\\  &  & -{\beta }_{n,i}^{j-1}{n}_{i}^{j-1}{)]}^{4}(({V}_{i}^{j-1}+{\rm{\Delta }}x\frac{{I}_{ion}}{C})-{V}_{K})\\  &  & +\delta {\bar{g}}_{Na}[{m}_{i}^{j-1}+{\rm{\Delta }}x({\alpha }_{m,i}^{j-1}\mathrm{(1}-{m}_{i}^{j-1})\\  &  & -{\beta }_{m,i}^{j-1}{m}_{i}^{j-1}{)]}^{3}[{h}_{i}^{j-1}+{\rm{\Delta }}x({\alpha }_{h,i}^{j-1}\mathrm{(1}-{h}_{i}^{j-1})\\  &  & -{\beta }_{h,i}^{j-1}{h}_{i}^{j-1})](({V}_{i}^{j-1}+{\rm{\Delta }}x\frac{{I}_{ion}}{C})-{V}_{Na})+R[{V}_{i}^{j-1}+{\rm{\Delta }}x\frac{{I}_{ion}}{C}]\\ {I}_{i}^{j+1} & = & \gamma {\bar{g}}_{K}[{n}_{i}^{j}+{\rm{\Delta }}x({\alpha }_{n,i}^{j}\mathrm{(1}-{n}_{i}^{j})\\  &  & -{\beta }_{n,i}^{j}{n}_{i}^{j}{)]}^{4}(({V}_{i}^{j}+{\rm{\Delta }}x\frac{{I}_{ion}}{C})-{V}_{K})\\  &  & +\delta {\bar{g}}_{Na}[{m}_{i}^{j}+{\rm{\Delta }}x({\alpha }_{m,i}^{j}\mathrm{(1}-{m}_{i}^{j})\\  &  & -{\beta }_{m,i}^{j}{m}_{i}^{j}{)]}^{3}[{h}_{i}^{j}+{\rm{\Delta }}x({\alpha }_{h,i}^{j}\mathrm{(1}-{h}_{i}^{j})\\  &  & -{\beta }_{h,i}^{j}{h}_{i}^{j})](({V}_{i}^{j}+{\rm{\Delta }}x\frac{{I}_{ion}}{C})-{V}_{Na})+R[{V}_{i}^{j}+{\rm{\Delta }}x\frac{{I}_{ion}}{C}]\end{array}$$

In order to account for spatial and temporal progression of the action potential, the system of coupled ODEs shown in equation (2) depicts a modified Hodgkin-Huxley Model to include another additional term, disregarded in all previous models, of form

$$\begin{array}{rcl}{J}_{i}^{j+1} & = & \frac{1}{2\pi rx+2\pi {(r)}^{2}}[\gamma {\bar{g}}_{K}{[{n}_{i}^{j}+{\rm{\Delta }}x({\alpha }_{n,i}^{j}(1-{n}_{i}^{j})-{\beta }_{n,i}^{j}{n}_{i}^{j})]}^{4}(({V}_{i}^{j}+{\rm{\Delta }}x\frac{{I}_{ion}}{C})-{V}_{K})\\  &  & +\delta {\bar{g}}_{Na}{[{m}_{i}^{j}+{\rm{\Delta }}x({\alpha }_{m,i}^{j}(1-{m}_{i}^{j})-{\beta }_{m,i}^{j}{m}_{i}^{j})]}^{3}[{h}_{i}^{j}+{\rm{\Delta }}x({\alpha }_{h,i}^{j}(1-{h}_{i}^{j})\\  &  & -{\beta }_{h,i}^{j}{h}_{i}^{j})](({V}_{i}^{j}+{\rm{\Delta }}x\frac{{I}_{ion}}{C})-{V}_{Na})+R[{V}_{i}^{j}+{\rm{\Delta }}x\frac{{I}_{ion}}{C}]]\end{array}$$

This novel additional term proposed and described by this paper quantifies the spatial as well as a temporal progression of the action potential, since *J* is treated as *J* = *J(i, j)* = *J(x*, *t)*.

By adding the term ${J}_{i}^{j+1}$, this paper, for the first time, describes both spatial and temporal propagation of not only the action potential in the form of depolarization and hyperpolarization of the neuronal membrane but also of an active spread of the axial current from the previous neural segment. This axial current then aids the action potential propagation and attenuation along the length of an axon and promotes the generation of an inhomogeneous electromagnetic field.

### Electromagnetic properties of cells make them susceptible to influences from electromagnetic fields generated by the axial and longitudinal current flow within the nerve fibers

After a new model which includes the spatio-temporal progression of an action potential, both in the form of ionic and axial currents, has been established, its influence on charged cells can be quantified. In this section we focused on spatio-temporal propagation of the action potential which induces a non-homogeneous time-varying electromagnetic field around the neurons which then exerts a force on the cells, therefore impacting their migration and adhesion.

Since cells are seen as negative point charges and the neuron can be modeled as a current carrying wire with periodic insulation in the form of the myelin sheath, in this paper we also propose that the nature of interaction of cells with the neurons and the myelin sheath expands beyond just chemical communication and includes the force that the electromagnetic field generated by a flowing current inside the neuron causes.

### New model of the action potential propagation through axial and longitudinal current flow precisely quantifies and describes the nature of time-varying electromagnetic fields generated around neurons

Even though the myelin itself insulates the axon and, therefore, prevents the spread of an electric field at those areas, magnetic flux can pass through a physical insulator and can, therefore, exert a force on the cells and impact their movement and, possibly, their activation. This means that, at the nodes of Ranvier, the cells are under an influence of an electromagnetic and at myelinated segments, only magnetic forces. Because the nerve fiber functions like a current-carrying wire, to obtain the magnetic field generated around the axon during the action potential propagation at the nodes of Ranvier, Biot-Savart Law for a straight wire was modified to include the spatial and temporal progression of the action potential and adapted to the notation used in the modified Hodgkin-Huxley model, yielding the Ampere-Maxwell equation. This magnetic field then, due to its non-homogeneity, induces an electric field.

To solve for $\overrightarrow{B}$, another system of coupled ODEs needed to be created, defining $\overrightarrow{I}$ as a function of space and quantifying *Q*_*enc*_.

$$(\begin{array}{rcl}{\oint }_{C}\overrightarrow{B}\cdot \overrightarrow{dl} & = & {\mu }_{0}\overrightarrow{I}+\frac{{Q}_{enc}}{dt}\\ \overrightarrow{I}(x,t) & = & \gamma {\bar{g}}_{K}[n(x-\mathrm{1)}+{\rm{\Delta }}t({\alpha }_{n}(x-\mathrm{1)(1}-n(x-\mathrm{1))}\\  &  & -{\beta }_{n}(x-\mathrm{1)}n(x-{\mathrm{1))]}}^{4}((V(x-\mathrm{1)}+{\rm{\Delta }}t\frac{{I}_{ion}}{C})-{V}_{K})\\  &  & +\delta {\bar{g}}_{Na}[m(x-\mathrm{1)}+{\rm{\Delta }}t({\alpha }_{m}(x-\mathrm{1)(1}-m(x-\mathrm{1))}\\  &  & -{\beta }_{m}(x-\mathrm{1)}m(x-{\mathrm{1))]}}^{3}[h(x-\mathrm{1)}\\  &  & +{\rm{\Delta }}t({\alpha }_{h}(x-\mathrm{1)(1}-h(x-\mathrm{1))}\\  &  & -{\beta }_{h}(x-\mathrm{1)}h(x-\mathrm{1))]((}V(x-\mathrm{1)}\\  &  & +{\rm{\Delta }}t\frac{{I}_{ion}}{C})-{V}_{Na})+R[V(x-\mathrm{1)}+{\rm{\Delta }}t\frac{{I}_{ion}}{C}]\\ {Q}_{enc} & = & f({g}_{K},{g}_{Na},{g}_{L},x,t)\end{array}$$

In this system of three coupled ODEs, we put forth the expression for the induced magnetic field, $\overrightarrow{B}$, generated by a temporal progression of the action potential and variability of ionic and axial currents, $\overrightarrow{I}$, through the axon with a spatio-temporal dependence of charge accumulation inside the neuron. In order for it to be solved, a precise measure of the cell’s charge and the charge enclosed within the neuron has to be made in the future.

Different from current models which approximated the current flow through the neuron as a continual flow of ions through sodium, potassium and leak channels which is constant in time during an action potential, here we have significantly upgraded understanding of this process and proposed an additional term of applied current which is to be added to each consecutive axonal segment. This results in a spatio-temporal progression of the current density in surrounding axonal elements.

Since the current in a neuron induces a time-varying magnetic field, the time-varying magnetic field, in turn, induces an electric field of non-electrostatic nature in a stationary conductor such as an axon.

This induced electric field is of the form

$$\overrightarrow{E}=\frac{1}{\mu \varepsilon }\int \overrightarrow{\nabla }\times \overrightarrow{H}dt$$

and it exerts a force on the moving cell in its vicinity.

Since $\overrightarrow{B}={\mu }_{0}\overrightarrow{H}$ and *ε* = *ε*_0_*ε*_*cns*_, then the electric field is

$$\overrightarrow{E}=\frac{1}{\mu {\varepsilon }_{cns}}\int \overrightarrow{\nabla }\times \overrightarrow{B}dt$$

### Electromagnetic forces exerted on cells are governed by the strength and direction of the electromagnetic field, the charge on the cell, its velocity and distance from the neuron

Since the cell is a negative point charge, then the force exerted on it by the induced electric field depends on, as expected by the hypothesis, the surface charge on a cell, $\overrightarrow{q}$, and the strength, magnitude and direction of the electric field, $\overrightarrow{E}$, as defined by Coulomb’s Law

$${\overrightarrow{F}}_{el}=q\overrightarrow{E}=\frac{q}{\mu {\varepsilon }_{cns}}\int \overrightarrow{\nabla }\times \overrightarrow{B}dt$$

where *q* is the charge on the cell and $\overrightarrow{E}$ is the induced electric field around the neurons.

Treating the axon as a current-carrying wire with periodic insulations in the form of the myelin sheath, the electric field can only exert a force on the cell at the regions of the nodes of Ranvier, where the axon is exposed to the extracellular matrix. On the other hand, at the insulated regions of the axon, it is the magnetic field that exerts a force on the cell moving in its vicinity of the form

$${\overrightarrow{F}}_{mag}=q\overrightarrow{v}\overrightarrow{B}sin\theta $$

where $\overrightarrow{v}$ is the velocity of the cell and *θ* is cell’s incident angle.

As both electric and magnetic forces on the cell are proportional to the strength of the electromagnetic field, the cell’s velocity and its surface charge, the greater the field strength, at distances closest to the neuron, and the greater the cell’s velocity and charge, the greater the force exerted on it by the electromagnetic field around the neurons.

The electromagnetic force changes value and direction around the axon and, dependent on the entrance angle of the cell, the force could not only modify the cell’s trajectory but also impact the ion flow through its membrane by altering its conformation and, subsequently, tweak the charge of the charged components on the plasma membrane. This would impact both the cell’s migration and adhesion abilities. Similar as for the quantification of the inhomogeneous magnetic field strength, the calculations for electric field strength and associated forces can only be done once the surface charge of the target cell has been measured.

### The range of the magnetic field and associated forces depends on its strength, direction and proximity of other charged particles

Even though the magnetic field induced by time-varying currents is inhomogeneous and varied in time, it can be viewed as homogeneous at small distances from the axon, making it significant for evaluating cells’ interactions with associated forces. With this, the maximum distance at which the field maintains its relative homogeneity, *D*_*max*_, and is of constant strength, is governed by the ionic, longitudinal and axial current, the length of the axonal segment and the thickness of the myelin sheath. Beyond this boundary, the inhomogeneity starts having an effect on the field’s strength which then falls off non-linearly with the distance.

#### Range of the magnetic field at the nodes of Ranvier

As there is no myelin sheath at the nodes of Ranvier, which are assumed to be of length *x* = *1*.0*8* ± *0.02* *μm*, the distance at which the electromagnetic field is of strength *3.0* × 10^−12^ *T* and still relatively homogeneous is

$${D}_{max}=6.606\,\mu m$$

This means that at distance of *6.606* *μm*, the magnetic field is still of the maximal strength and homogeneity and then falls off with distance and becomes increasingly inhomogeneous, spreading towards the scalp.

#### Range of the magnetic field at the myelinated segment of the fiber

Even though magnetic flux can penetrate right through the myelin sheath, the magnetic field falls off with a square of distance from the axon so, even though the initial field is as strong as it is at the nodes of Ranvier, the strength that the cells detect right at the surface of the myelin sheath is weaker than it is at the nodes of Ranvier.

With this, the distance from the axon, covered by the myelin sheath, at which the electromagnetic field acts upon is

$${r}_{max}=5.696\,\mu m$$

As the thickness of the myelin sheath, on average, is *d* = *3.63* ± *0.05* *μm*, and *D*_*max*_ = *r*_*max*_ − *d*, the distance from the surface of the myelin sheath over which the electromagnetic field is still homogeneous is

$${D}_{max}=2.066\,\mu m$$

This distance represents the distance from neurilemma on which the cells can still feel a homogeneous, constant magnetic field.

#### Range of the magnetic field around nerves and nerve tracts

In above mentioned calculations, we started from one nerve fiber which consists of one axon and its myelin sheath. In reality, nerve fibers always come in bundles which then form nerves and nerve tracts. In order to model an average nerve tract composed of 100 axons, the distance between individual axons within the nerves and tracts is treated as negligible. Therefore, the whole nerve is simplified as a wire with a radius R, corresponding to the radius of the nerve or the tract, up to neurilemma. In this case the range of the field is

$${D}_{max}=12.005\,\mu m$$

### Strength of the magnetic field as a function of the distance from the node of Ranvier or the myelin sheath

**Figure 4:**
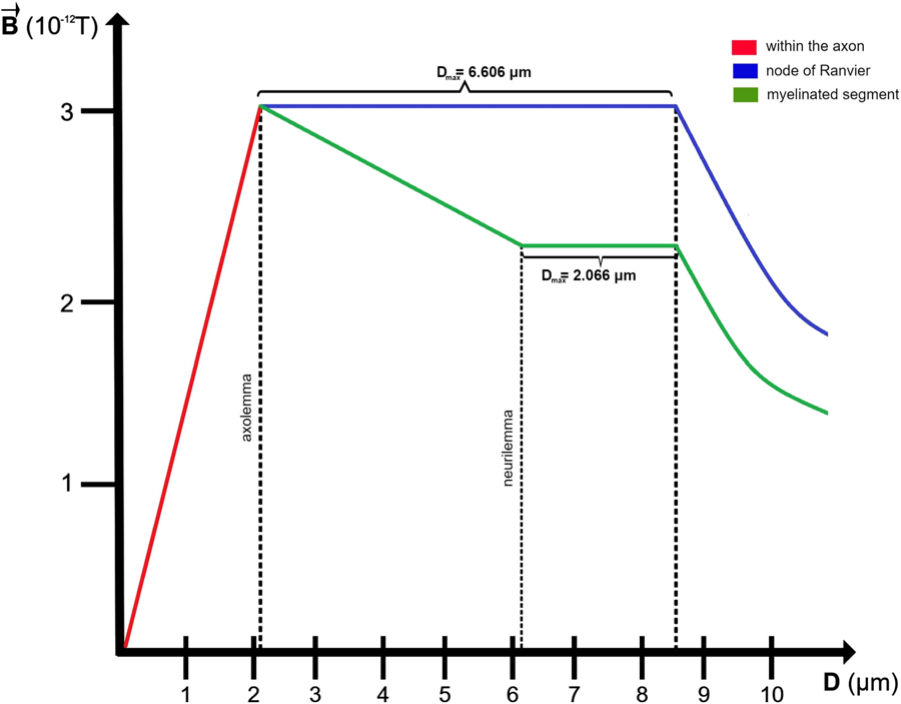
Strength of the magnetic field as a function of distance from the center of an axon. At myelinated segments, the magnetic field reaches its maximum of 3.0 × 10^−12^ *T* at axolemma, the outer membrane of the axon, then falls off to 2.3 × 10^−12^ *T* once it reaches neurilemma, the outer layer around the myelin sheath. From neurilemma, it keeps its homogeneity for distance of 2.066 *μm* and then it starts to decay. On the other hand, at nodes of Ranvier, the magnetic field stays homogeneous up to distances of 6.066 *μm* away from axolemma. After this the field starts decaying non-uniformly with distance.

As the electromagnetic field is a product of a non-uniform current distribution and the spatio-temporal propagation of the action potential, at non-myelinated segments, nodes of Ranvier, the field itself is highly inhomogeneous at distances bigger than *6.606* *μm* from axolemma, the outer sheath of an axon. On the other hand, at the myelinated segments, the field falls off in strength between axolemma and the surface of the myelin sheath, neurilemma, and becomes homogeneous only at neurilemma, reaching distances of *2.066* *μm*. Even though the field’s strength falls of with distance, the relationship between the magnetic field strength and range is highly linear until *r* = *D*_*max*_, and only starts falling off non-linearly once $r > {D}_{max}$ (Fig. 4).

As seen in Fig. 4, the decay rate and maximum value of the electromagnetic field highly depend on whether the region is myelinated or unmyelinated. At the nodes of Ranvier, which are unmyelinated regions, the magnetic field reaches strengths up to

$$\overrightarrow{B}=3.0\times {10}^{-12}T$$

which then remains constant and relatively homogeneous up until *6.606* *μm* away from the surface of the node of Ranvier, axolemma.

At the myelinated regions, the magnetic field reaches strengths up to *3.0* × 10^−12^ *T* but it starts decaying right after axolemma to values of

$$\overrightarrow{B}=2.3\times {10}^{-12}T$$

After reaching this constant value, the magnetic field is homogeneous and uniform up to *2.066* *μm* from neurilemma, after which it starts decaying in the same manner as the field around nodes of Ranvier.

Going past distances of *D*_*max*_, the magnetic fields described by this paper start exhibiting inhomogeneities and spatio-temporal variations and can no longer be simply plotted. Actually, these same fields can be detected by MEG but, because of the distance from the source and the inhomogeneity of the field, are then measured with strengths reaching only 10^−15^ *T*.

### Strength of the magnetic field around nerves and tracts

In order to quantify the exact strength of the magnetic field around nerves and tracts, we again took an average size of the bundle to be composed of 100 axons. In that case,

$$\overrightarrow{B}=6.0\times {10}^{-12}T$$

As it can be noted, the maximum strength of the magnetic field only doubled from the magnetic field strength for an individual axon. This is due to current dissipation and magnetic-field-component cancellation caused by different functional axons, within the same tract or nerve, carrying opposing directional currents.

### The effect of demyelination on the magnetic field strength and range

In pathogenic conditions, as the myelin sheath has started degenerating, the thickness of the myelin sheath will impact the distance upon which the magnetic field can act. The more the myelin sheath will degenerate, the greater the distance from the neuron on which the electromagnetic field can be felt by the cells.

As the myelin sheath will begin to deteriorate, the magnetic field strength acting upon the cells will increase from 2.3 × 10^−12^*T* to close to 3.0 × 10^−12^*T* and more. This means that a greater force will be felt by the cells at the myelinated regions as opposed to the nodes of Ranvier since the magnetic field will be acting upon greater distances.

Depending on the pattern of demyelination, the sum electromagnetic field around the neurons could also significantly alter and either increase, as a result of changing directions of surrounding field components, or decrease and be completely canceled by oppositely directed field components. With this, a 50% demyelination of the myelinated segments will result in a increase in the magnetic field strength by 0.35 × 10^−12^*T* whilst a 100% demyelination will result in an increase up to 0.7 × 10^−12^*T*.

## Discussion

In this work we developed a new model which describes the interaction of electromagnetic fields around axons and negatively charged cells. By proposing a spatio-temporal modification to the existing Hodgkin-Huxley Model of an axon, which includes the evolution of ionic and axial currents and voltages during the propagation of an action potential in the term ${J}_{i}^{j+1}$, this work allows for exploration of the novel geometry and the source of electromagnetic fields in the CNS and calculation of their strength and direction around nerve fibers. As the Hodgkin Huxley neuron model is the most computationally expensive model of a neuron’s function, we estimated that our addition of ${J}_{i}^{j+1}$ increases the computation cost by 20–40%, depending on the software and hardware used to perform the computations in question.

Instead of using the Biot-Savart Law for constant magnetic field, here we propose an application of the Ampere-Maxwell Equation for the time-varying and inhomogeneous electromagnetic fields, which are a consequence of the time-dependent current generated by action potential propagation, at distances greater than *2.066* *μm* from neurilemma, at the myelinated segments, and *6.606* *μm* from axolemma, at the nodes of Ranvier. What is different between ours and the previous models is that, while in their models the applied current is the current that is applied onto an axon in the form of a one-time impulse to initiate action potential propagation, the applied current term in our model becomes the axial current. Approximating that there is no intracellular longitudinal current flow past the given initial impulse, Hodgkin and Huxley oversimplified the model by only including the applied current term in the first spatial iteration of the current.

Our model reveals that, with such interconnectedness between intracellular, extracellular and ionic current flow, residual current flow, which we named the “axial current’, does, indeed, flow from the previous to consecutive axonal segment and plays a role in attenuating and aiding action potential propagation and initiation. Our model also includes the existing displacement currents, area and free current density as well as magnetic permeability and electric permittivity of the nerve tissue. Here we have shown that the nerve fiber can be modeled as a current-carrying wire whose current strength varies according to the relative position of the node of Ranvier or the myelinated segment and associated action potential propagation times. If one such region is observed, the current flow through the neuron induces a time-varying circular magnetic field of localized strengths up to 3.0 × 10^−12^*T* which, in turn, induces an electric field, both of which exert a force on a cell in its vicinity.

Interestingly, the fields that are described in this paper are, actually, the same fields detected by MEG. The reason why MEG^14^ detects fields of 10^−15^ *T* is not only the distance from the source but also their inhomogeneity, which our model precisely describes. Thus, our model, in much more detail, quantifies the electromagnetic fields in the nervous system and explains the nature of the signal detected by MEG. With this, both the electromagnetic fields described by our model and the magnetic field detected by MEG, are valid and interconnected. One is observing the micro values of the electromagnetic field strength at levels of cells and single axons, such as described by our model, and the other detects the macro-values of the magnetic field strength, such as seen on MEG.

Since our model suggests interactions of cells and tissue based on electromagnetic fields, our theory also puts forth the concept of “electromagnetic receptors” present on cells. These receptors would enable them to recognize electromagnetic fields in their surrounding by either responding to the general presence of an EM field or to its strength, scope and changes from the starting healthy state. Based on discoveries in the recent decade, it is necessary to take in account that interactions between EM fields and cells both influence their adhesion to the fibers, ECM or other cells, as well as the dynamics of possible immune reaction^9,10^. In addition, it was also recently shown that cell charge complements the cell’s activity with respect to the surface charge of the target cell^10^. In another approach which attempted to influence the immune reaction, “immunocamouflage” of cells lead to significant changes in reactivity of lymphocytes^12^. Finally, in their detailed analyses, Kyluik and Scott explained how at least part of interactions between lymphocytes and target cells are modulated over precise quanta of charge^11^, showcasing the importance of cell charge in regulating its activity and, with that, its motion and activation around the inhomogeneous time-varying electromagnetic fields proposed by our work.

Taking into account the radius and distance terms which contribute to the electromagnetic field strength equations, here we have shown that the strength of the magnetic field reaches distances of *D*_*max*_ = *6.606* *μm* at the nodes of Ranvier and *D*_*max*_ = *2.066* *μm* at the myelinated segments, where *D*_*max*_ is the distance of the cell from the surface of the nerve fiber. As the myelin sheath thickness and consistency change during onset and progression of majority of pathologic events, the electromagnetic fields associated with those myelinated fibers change in direction, value and location. Such would be the example of a 50% demyelination of an individual myelinated segment which will result in a increase in the magnetic field strength by 0.35 × 10^−12^*T*, yielding a magnetic field of 3.35 × 10^−12^*T*. On the other hand, a 100% demyelination of a particular axonal segment will result in an increase up to 0.7 × 10^−12^*T*, resulting in a magnetic field strength up to 3.75 × 10^−12^*T*. These changes not only then influence impulse propagation but also further degeneration due to newly-generated electromagnetic imbalance at that region.

Next, with our theory, we also propose that electromagnetic fields in pathological conditions, due to degeneration of the nerve fiber or myelin sheath destruction, yield a state of electromagnetic field imbalance. The created field imbalance then contributes to the detection of existing electromagnetic fields by cells in their vicinity, and their consequent adherence to axonal fibers and the myelin sheath through the proposed electromagnetic receptors on cells surface or some other, still unknown, detecting mechanism. In order to prove this concept, we have conducted an initial study in the form of an *in-vivo* MRI cell tracking experiment using mouse T-lymphocytes and a mouse stroke model. The results from this experiment have clearly shown that, as opposed to T-lymphocytes with intact surface charge, which migrate to the lesion, uncharged T-lymphocytes remain at the injection site 3–7 days after the treatment (*data obtained in this study are not shown*). Here, the proposed mechanism by which the cells’ surface charge dictates its motion within the CNS follows the Law of Charges and the Lorentz Force Law. These laws state that, when a charged particle is moving through an electromagnetic field, as the one existing around neurons, it feels a force, F, being exerted on it. This force then affects the migration trajectory and the speed of the charge within the field in question. Since T-lymphocytes can be seen as negative point charges under the influence of inhomogeneous electromagnetic fields around neurons, their migration, on top of being dictated by the intrinsic and external biological mechanisms, is impacted by the electromagnetic force acting upon them as well. Governed by the same laws is also the idea that electromagnetic fields do not exert any force on an uncharged particle, which is, in this case, the treated T-lymphocyte. This indicates that, if the electromagnetic force no longer has an impact on the uncharged cell’s trajectory in the CNS, changing of the cell’s external charge could be a valuable therapeutic in diseases where the immune response exhibits abnormal function or is needed for additional tissue repair.

By applying this theory to some of the most common pathological conditions in the nervous system, which, by the way, represent some of the most common human diseases in general, this paper proposes a role of electromagnetic fields in ischemic stroke and multiple sclerosis, among other diseases, where cells initiate or exaggerate the injury by initiating secondary negative effects of inflammation. Once cells penetrate the blood brain barrier, which is a common event in all neuroinflammatory diseases, negatively charged cells start to enter the range of electromagnetic fields around neurons, which then change the cell’s migration and adhesion pattern in a way that corresponds to the nature of electromagnetic forces exerted on them. The strength, direction and magnitude of these forces are then determined by the ionic and axial current flow within that segment as well as the displacement current, defined by the rate of change of electric displacement. Depending on the initial trajectory of the cell, once it is in the range of the electromagnetic field, it will either be deflected or enter to close proximity of the nodes of Ranvier and the myelin sheath and, correspondingly, change its migration or adhesion pattern. Moreover, the interactions of T-lymphocytes with the myelin sheath are a well known basis for multiple sclerosis, one of the most common autoimmune diseases, in which the cause of such a sudden affinity of T-lymphocytes for the myelin sheath and the pattern of their distribution throughout CNS are still unknown and could, potentially, begin to be explained by our theory.

On that account, in one of the recent publications by Lukens *et al*. on pathogenesis of multiple sclerosis, it has been shown that a mutation in the gene NLRP12 causes the cells to go haywire^15^. As much as this suggests the cause behind the T-lymphocyte’s penetration through the blood brain barrier, it does little to explain the process behind the diseases onset and the forces between the myelinated axons and cells that make them recognize myelin as an antigen. In their work, Lukens *et al*. have proven that multiple sclerosis starts off when the protein produced by the mutated NLRP12, which usually acts as a brake for cells by controlling their response to inflammation, disrupts the natural process and provokes severe inflammation^15^. This inflammation, then, increases the permeability of the blood-brain barrier, allowing the white blood cells to infiltrate into the CNS and destroy the myelin sheath. However, none of this explains why exactly the cells recognize myelin as an antigen and start the autoimmune response. If myelin did, indeed, activate the cells, then the C-type postganglionic and dorsal root nerve fibers in the autonomic nervous system and dorsal root ganglia, respectively, that are unmyelinated, would be unaffected by multiple sclerosis – and that is not the case. With this, as an alternative to myelin presenting an antigen that onsets the cell activation in the CNS in patients with multiple sclerosis, we propose that cells could possess a mechanism for recognizing the change in natural electromagnetic fields around neurons and respond to it.

Because the myelin sheath is an insulator, no electric field can be detected by the cells outside of the axon but a magnetic flux can pass right through it and, therefore, generate a magnetic field right outside the myelinated axonal segment. As opposed to the periodic insulations along the axon, the nodes of Ranvier boast an exposed axon surrounding which is both an electric and a magnetic field component. If the concept of “electromagnetic receptors” that we present in this paper is to be proven, it would suggest that the reason the cells initiate the immune response for the destruction of the myelin sheath is because they recognize the existence of a non-uniform magnetic field around the myelinated segments and, in accordance with laws of physics, no changing magnetic field created in our body can be isolated from an electric field. Since every non-uniform time-varying magnetic field in nature generates an induced electric field, the “electromagnetic receptors” could recognize the non-existence of a detectable electric field, which is shielded by the myelin sheath, as a sign of an external pathogen that is harmful to the normal workings of our body and is impacting the functionality of the CNS.

On the other hand, the concept of “electromagnetic receptors” brings along the idea of detection of electromagnetic field strength and direction change and the consequences it would have on cells mobility, activity and adhesion. If, for example in ischemic stroke, the cells were to respond to the initiated immune sequence, they would also be able to recognize the lesioned hemisphere or region by recognizing the change in electromagnetic fields from the healthy condition in the surrounding tissue. As opposed to the healthy tissue, lesioned tissue will include demyelinated and degenerated nerve fibers which will, no longer, be able to conduct electricity or correctly aid action potential propagation. This change in current flow would then cause a change in the induced electromagnetic field in the form of alterations in its value and direction or even changes in the overall existence of such fields. If a neuron is to be degenerated to such an extent that it can no longer conduct impulses, no electromagnetic field will be detected around that region and, with that, the region will be seen, again, as an external pathogen and the cells will initiate a response to aid its regeneration or cause further damage.

Ultimately, the theoretical basis of our new model can be found in works by Kirschiving *et al*. and Nordenstrom *et al*. In their paper titled “Magnetite biomagnetization in the human brain”, Kirschivink *et al*. described that throughout the CNS, “white blood cells behave like magnetic microparticles in plasma”^12^, suggesting that electromagnetism has an active role in immune responses. The idea that the cells themselves carry a magnetic property implies that they, themselves, are susceptible to be influenced by electromagnetic forces generated by the EM fields around neurons and should then be evaluated for any influence EM fields can have on their migration, adhesion and activation. Moreover, in his book on Biologically Closed Electric Cirtuits (BCEC), Nordenstrom hypothesized that, once a microorganism finds its way into the body, it starts producing an electric field^16^ which then modifies the function of the immune system by attracting the white blood cells to the site. Applying a similar idea to tumor cells in breast and lung cancer, Nordenstrom showed that “electronegative” white blood cells are “attracted to the tumor during its phase of electropositive polarization as an electrophoretic process within the Vascular-Interstitial Closed Circuit (VICC)”^16^. Working off their theories, our model aimed to explain the role of cell charge in its migration, adhesion and activation once it has, in a pathological condition, penetrated the blood brain barrier and come in contact with the non-uniform time-varying electromagnetic fields around axons.

By proposing the existence of an inhomogeneous time-varying electromagnetic field around nerve fibers, our model opens the possibility of novel potential therapeutic approaches based on interactions between electromagnetic fields and negatively charged cells. By finding a way to modulate the surface charge on cells, whilst still maintaining their “functionality”, their migration and adhesion could be influenced or programmed to solely rescue and not exaggerate the injury site. In one such attempts it was recently shown that incorporation of the chondroitin sulfate in the membrane of bacterial cell model changed the surface charge and behavior of cells^17^. Another potential approach would be targeting frequency, strength and direction of the magnetic fields. If the nature of the electromagnetic field around a neuron is defined, in a healthy state, as the presence of electromagnetic fields around the nodes of Ranvier and magnetic fields around the myelinated segments of strength *B*_1_ then, in a pathological condition where the myelin sheath and the neuron have started degenerating, the direction and frequency of the magnetic field will change as the myelin sheath is degenerated, resulting in change in strength into *B*_2_. This will then result in appearance of the electric field even in the myelinated regions, as the myelin sheath will have started deteriorating and will no longer be a perfect insulator. In such a case, we hypothesize that applying precise external electromagnetic fields, in the form of a helmet applied to the head with localized EM field transmitters directed towards targeted regions, could influence the interaction between cells and innate electromagnetic fields within the CNS, and assist in repairing the damaged tissue. Finally, an alternative approach based on using combination of ultrasound opening of the blood brain barrier and magnetic guidance of cells into the CNS, exploiting their electromagnetic affinity, could also be used as it has been increasingly exploited and already proven as feasible^18^, with alterations proposed in this work in the form of cell’s surface charge modification. Whether it be with targeting of cell’s surface charge to modify their functionality within the CNS, direct them to the target site or with application of localized EM field transmitters, we believe that such approaches would not only enable us to treat neuroinflammatory and neurodegenerative diseases but also modulate their onset and improve their pathogenesis and should, therefore, be explored further.

## Methods

### Crank-Nicholson Method for solving the Hodgkin Huxley model of an axon

As Hodgkin-Huxley Model starts off, the ion gates in the cell, and neuronal membrane, are controlled by the potential across the membrane and are, thusly, known as voltage gated channels. According to Hodgin-Huxley model, these channels can be described by a two-state Markov process, where O denotes the opening and C the closing of the voltage gated channels:

$$C\underset{{\rm{k}}-}{\overset{{\rm{k}}+}{\rightleftarrows }}O$$

Here, according to the two-state Markov process definition, fraction of open channels can be denoted by f_0_, *k* ^+^ is the rate constant of opening and *k*^−^ the rate constant of the closing of the voltage gated channels.

$$\frac{d{f}_{0}}{dt}=-\,{k}^{-}{f}_{0}+{k}^{+}(1-{f}_{0})=\frac{{f}_{\infty }-{f}_{0}}{\tau }$$

Where f_∞_ is defined by

$${f}_{\infty }=\frac{{k}^{+}}{{k}^{+}-{k}^{-}}$$

and the time constant, *τ*, describing the opening and closing of activation gates depends on rate constants for open/closed channel transitions:

$$\tau =\frac{1}{{k}^{+}-{k}^{-}}$$

Since the ion channels are charged amino acid side chains on the proteins, what will, ultimately, influence the open/closed state transition rate is the potential difference across the membrane. With this, the rate constants are expected to have the Arrhenius-like exponential form for rate constants.

$${k}^{+}={k}_{0}^{+}{e}^{-\alpha V},\,{k}^{-}={k}_{0}^{-}{e}^{-\beta V}$$

*α* and *β* are activation and inactivation constants associated with opening and closing of the voltage gated ion channels, respectively.

As the voltage in the circuit, in this case, depends on the activation and transition rates of the gates and ion channels, then the initial voltage in the neuronal circuit described by Hodgkin-Huxley model of an axon is

$${V}_{0}=\frac{1}{\beta -\alpha }ln(\frac{{k}_{0}^{-}}{{k}_{0}^{+}})$$

The biggest input Hodgkin-Huxley model had in this case was the recognition that the conductance of each ion channels depends on both activation and inactivation gates whose number, and probability of them being open or closed, depends on the nature of the ionic channel^19^.

By recognizing the importance of activation and inactivation gates, Hodgkin and Huxley proved that the potassium channel depends on four activation gates and the sodium channel depends upon three activation and one inactivation gate, denoted by the power superscipts. These activation and inactivation gates are designated by m, n and h, which are dimensionless quantities between 0 and 1. *n* is the probability of the potassium activation gate being open, *m* is the probability of the sodium activation gate being open and *h* is the probability of the sodium inactivation gate being open. From here, the conductance of each respective ion channels, *g*, can be written as a function of the average conductance, $\bar{g}$, and the respective probabilities of activation and inactivation gates.

3 $${g}_{K}={\bar{g}}_{K}{n}^{4},\,{g}_{Na}={\bar{g}}_{Na}{m}^{3}h$$

As each neuron has multiple ion channels that all, in some lesser form, contribute to the overall membrane potential and, with that, the net electricity and electromagnetic fields, contributions from other channels are taken into account through non-specific ionic leak currents, denoted by a subscript L.

4 $${g}_{L}={g}_{L}(V)$$

Since potassium, sodium and other ion gates can be controlled by the membrane potential and, in return, play a role in establishing the membrane potential, they are all said to be functions of V:

$${g}_{K}={g}_{K}(V),\,{g}_{Na}={g}_{Na}(V),\,{g}_{L}={g}_{L}(V)$$

Because n, m and h are associated with potassium channel activation, sodium channel activation and sodium channel inactivation, respectively, as seen in equations (3) and (4), they are of the form:

$$\frac{dm}{dt}=\frac{{m}_{\infty }(V)-m(V)}{{\tau }_{m}(V)}$$

$$\frac{dn}{dt}=\frac{{n}_{\infty }(V)-n(V)}{{\tau }_{n}(V)}$$

$$\frac{dh}{dt}=\frac{{h}_{\infty }(V)-h(V)}{{\tau }_{h}(V)}$$

where the value of *τ*_*p*_, whose subscript is defined as *p* = *(n, m, h)*, depends on voltage and position but not time. Then *τ*_*p*_ can be defined as

$${\tau }_{p}(V)=\frac{1}{{\alpha }_{p}(V)+{\beta }_{p}(V)}$$

Here, p can be interchanged for n, m or h to account for sodium or potassium channel activation or inactivation. To account for i-th ion channels, Hodgkin-Huxley introduced *α*_*p*_(*V*) and *β*_*p*_(*V*) as the rate constants, which depend on voltage but not time. This step, necessarily, takes away the temporal component from their circuit model of an axon and will be addressed in our model by addition of the *I*_*ax*_ term further along. For each probability of ion channel activation or inactivation, the rate constants are as follows:

$${\alpha }_{m}(V)=\frac{\mathrm{0.1(}V+\mathrm{40)}}{1-{e}^{-\mathrm{0.1(}V+\mathrm{40)}}},\,{\beta }_{m}(V)=4.0{e}^{-\mathrm{0.00556(}V+65)}$$

$${\alpha }_{n}(V)=\frac{\mathrm{0.1(}V+\mathrm{55)}}{1-{e}^{-\mathrm{0.1(}V+\mathrm{55)}}},\,{\beta }_{n}(V)=0.125{e}^{-\mathrm{0.0125(}V+\mathrm{65)}}$$

$${\alpha }_{h}(V)=0.07{e}^{-\mathrm{0.05(}V+\mathrm{65)}},\,{\beta }_{h}(V)=\frac{1}{1+{e}^{-\mathrm{0.1(}V+\mathrm{35)}}}$$

Here, each of the numerical constants following the rate constants have been experimentally measured and obtained by Hodgkin-Huxley in their 1952 paper titled “A quantitative description of membrane current and its application to conduction and excitation in nerves”^19^.

Since an electromagnetic field is generated as a byproduct of stationary charges and moving currents during an action potential within an axon, the resulting current that yields the non-uniform time-varying electromagnetic field proposed by our theory is a sum of the capacitive, I_*cap*_, and the ionic current, I_*ion*_, as seen in Fig. 1, by the Hodgkin Huxley model,  and the additional terms of axial, I_*ax*_, and longitudinal, I_*lng*_, intracellular currents propagating alongside the action potential, put forth by out model. As their names state, the capacitive current occurs due to the capacitance of the cell and the ionic current occurs due to the ion flow across the cell membrane. Both the capacitive and ionic currents from the Hodgkin-Huxley Model account for the cycles of hyperpolarization and depolarization of the neural membrane through ion influx and outflux but, due to the ionic movement in the extracellular space and the arrangement of charge in both intracellular and extracellular space during action potential propagation, we propose an addition to the Hodgkin-Huxley Model in the form of afore mentioned axial and longitudinal currents which would, more accurately, describe and quantify the facilitation of action potential propagation.

Whilst the capacitive current is the change in membrane voltage multiplied by the cell’s capacitance,

$${I}_{cap}=C\frac{dV}{dt}$$

the ionic current is the sum of the individual current flows over all the ion channels.

$${I}_{ion}=\sum {I}_{i}$$

On the other hand, the longitudinal current proposed by our theory, is the result of created and applied potential difference due to the passive spread of action potential and subsequent transversal ionic current flow.

$${I}_{lng}=\frac{V}{R}$$

Axial current is then the sum of longitudinal current and fractions of capacitive and ionic currents that initiate action potential propagation in the following axonal segment.

According to the Hodgkin-Huxley model, the ionic current is equal to the conductance of the ion channel multiplied by the driving force across the membrane, including sodium, potassium and leak ionic currents.

$${I}_{ion}=\sum {I}_{i}=\sum {g}_{i}(V-{V}_{i})$$

$${I}_{ion}={g}_{K}(V-{V}_{K})+{g}_{Na}(V-{V}_{Na})+{g}_{L}(V-{V}_{L})$$

Calculating the derivatives using Euler’s first order approximation for the evolution of voltage gating variables n, m, h and V, the expressions become:

$$V(i+\mathrm{1)}=V(i)+{\rm{\Delta }}t\frac{{I}_{ion}}{C}$$

$$n(i+\mathrm{1)}=n(i)+{\rm{\Delta }}t({\alpha }_{n}(i\mathrm{)(1}-n(i))-{\beta }_{n}(i)n(i))$$

$$m(i+\mathrm{1)}=m(i)+{\rm{\Delta }}t({\alpha }_{m}(i\mathrm{)(1}-m(i))-{\beta }_{m}(i)m(i))$$

$$h(i+\mathrm{1)}=h(i)+{\rm{\Delta }}t({\alpha }_{h}(i\mathrm{)(1}-h(i))-{\beta }_{h}(i)h(i))$$

If the diameter of the neuron is *d*, its circumference is *π d* and the surface of a piece of step length is Δ*x*, then the capacitance of this piece of neuron is *C* = *πd*Δ*xc*. Here, *c* is the specific capacitance per unit area.

According to Kirchoff’s Law, the sum of all the voltages around a loop of the circuit has to be equal to zero. In this case, the capacitive and the ionic leak currents have to be equal in order to describe a steady state. Thus, it can be written that:

5 $$C\frac{dV}{dt}={g}_{K}(V-{V}_{K})+{g}_{Na}(V-{V}_{Na})+{g}_{L}(V-{V}_{L})+{I}_{app}$$

where *I*_*lng*_ is the applied current - defined by the Hodgkin-Huxley Model as the current generated by the voltage step applied to the neuron.

To account for the change in membrane capacitive and ionic potential along the neuron, the Hodgkin-Huxley Model first needs to be solved in a compartmental method manner for spatio-temporal propagation of the action potential. This is done by using ODEs and adding additional coupling terms to relate the spatial and temporal dependence between different neuronal compartments.

The most common ways of solving such ODEs is by using forward-Euler or backward-Euler Methods but they both, with themselves, carry numerical instability. To surpass this problem, a Crank-Nicholson Method is used. With the combination of the forward- and backward- Euler Method and their averaging, the Crank-Nicholson Method enables dispelling of numerical instabilities and increases spatial and temporal fidelity.

$${C}_{m}\frac{{V}_{i}^{j+1}-{V}_{i}^{j}}{{\rm{\Delta }}t}=\frac{1}{2}(\frac{d}{4{R}_{i}}\frac{{V}_{i+1}^{j+1}-2{V}_{i}^{j+1}+{V}_{i-1}^{j+1}}{{\rm{\Delta }}{x}^{2}}-\,{G}_{m}{V}_{i}^{j+1}-\,{J}_{i}^{j+1}+\frac{d}{4{R}_{i}}\frac{{V}_{i+1}^{j}-2{V}_{i}^{j}+{V}_{i-1}^{j}}{{\rm{\Delta }}{x}^{2}}-\,{G}_{m}{V}_{i}^{j}-{J}_{i}^{j})$$

### Determining the resistance and area of a neuron

Modeling the neuron as a current carrying wire, we state that the electrical resistance of a wire is greater for a longer wire, lesser for a wire of larger cross-sectional area and depends on the material of which the wire is made. This is all accounted for in the resistivity, *ρ*. Knowing that

$$R=\frac{\rho L}{A}$$

then we can say that, for now, the length of the axonal segment *L* is equal to the step-size *x*. The area of the neuron of surface length *x* is

6 $$A=2\pi \frac{d}{2}x+2\pi {(\frac{d}{2})}^{2}=2\pi rx+2\pi {(r)}^{2}$$

With this, the change in resistance of the neuron after a step-size of *x* becomes

7 $$R=\frac{\rho x}{2\pi r(x+r)}$$

### Derivation of the longitudinal current term and its solution using the Crank Nicholson Method

Because the initial longitudinal current corresponds to a voltage spike within that neural segment, divided by the resistance of the neural fiber, ${I}_{lng}=\frac{V}{R}$, to obtain the value of the longitudinal current and its corresponding temporal propagation along the length of a neuronal segment, *x*, resistance of the axon, R, had to be obtained, modeled like a wire.

Since we are interested in temporal propagation of the applied current then

$$\frac{d{I}_{lng}}{dt}=\frac{d}{dt}(\frac{V}{R})$$

and, approximating that within the time scale it takes for AP to propagate, the resistance of a neuron is not a function of time, the equation becomes

$$\frac{d{I}_{lng}}{dt}=R\frac{dV}{dt}$$

To solve this equation we, again use the Euler Methods to obtain

$${I}_{lng}={I}_{lng,i}^{j}=R[V(j-\mathrm{1)}+{\rm{\Delta }}x\frac{{I}_{ion}}{C}]$$

With this, the axial current density, the sum density of longitudinal and fractions of ionic and capacitive currents, $\overrightarrow{J}$, can now be rewritten using temporal and spatial indexes seen in the Crank-Nicholson Method by substituting each of these terms into equation (5). To obtain ${J}_{i}^{j+1}$ and ${J}_{i}^{j}$, forward and backward Euler Methods need to be applied and a function for both variables needs to be derived.

Following the Euler Method, we can rewrite the axial current as two separate equations corresponding to ${I}_{i}^{j+1}$ and ${I}_{i}^{j}$ using temporal derivatives of probabilities of ionic activation and inactivation gates being open or closed.

8 $$\begin{array}{rcl}{I}_{i}^{j} & = & \gamma {\bar{g}}_{K}[n(j-\mathrm{1)}+{\rm{\Delta }}x({\alpha }_{n}(j-\mathrm{1)(1}-n(j-\mathrm{1))}\\  &  & -{\beta }_{n}(j-\mathrm{1)}n(j-{\mathrm{1))]}}^{4}((V(j-\mathrm{1)}+{\rm{\Delta }}x\frac{{I}_{ion}}{C})-{V}_{K})\\  &  & +\delta {\bar{g}}_{Na}[m(j-\mathrm{1)}+{\rm{\Delta }}x({\alpha }_{m}(j-\mathrm{1)(1}-m(j-\mathrm{1))}\\  &  & -{\beta }_{m}(j-\mathrm{1)}m(j-{\mathrm{1))]}}^{3}[h(j-\mathrm{1)}\\  &  & +{\rm{\Delta }}x({\alpha }_{h}(j-\mathrm{1)(1}-h(j-\mathrm{1))}\\  &  & -{\beta }_{h}(j-\mathrm{1)}h(j-\mathrm{1))]((}V(j-\mathrm{1)}\\  &  & +{\rm{\Delta }}x\frac{{I}_{ion}}{C})-{V}_{Na})+R[V(j-\mathrm{1)}+{\rm{\Delta }}x\frac{{I}_{ion}}{C}]\end{array}$$

9 $$\begin{array}{rcl}{I}_{i}^{j+1} & = & \gamma {\bar{g}}_{K}{[n(j)+{\rm{\Delta }}x({\alpha }_{n}(j\mathrm{)(1}-n(j))-{\beta }_{n}(j)n(j))]}^{4}\\  &  & \times ((V(j)+{\rm{\Delta }}x\frac{{I}_{ion}}{C})-{V}_{K})+\delta {\bar{g}}_{Na}[m(j)\\  &  & +{\rm{\Delta }}x({\alpha }_{m}(j\mathrm{)(1}-m(j))-{\beta }_{m}(j)m(j{))]}^{3}[h(j)+{\rm{\Delta }}x({\alpha }_{h}(j\mathrm{)(1}-h(j))\\  &  & -{\beta }_{h}(j)h(j))]((V(j)+{\rm{\Delta }}x\frac{{I}_{ion}}{C})-{V}_{Na})+R[V(j)+{\rm{\Delta }}x\frac{{I}_{ion}}{C}]\end{array}$$

Both of these expressions presented in equations (8) and (9) serve to define and quantify the axial current at both the previous and the subsequent axonal segments and its temporal progression.

### Modeling of the electromagnetic field around neurons generated by the passing axial, intracellular and ionic currents

Even though the current within an axon could be approximated as steady in time over a small enough, Δ*x*, element of a neuron, and would then signify that the induced magnetic field does not vary in time, the propagation of action potential is encoded with sinusoidal threshold properties. The sinusoidal frequency of the action potential then induces a time-varying magnetic field and a new source term has to be added to the Biot Savart Law called displacement current, resulting in the Ampere-Maxwell equation.

10 $$\overrightarrow{\nabla }\times \overrightarrow{B}={\mu }_{0}\overrightarrow{J}+{\varepsilon }_{0}\frac{\partial \overrightarrow{E}}{\partial t}$$

Where $\overrightarrow{B}$ is the magnetic field, $\overrightarrow{J}$ is the total current density, $\overrightarrow{E}$ is the induced electric field, *μ*_0_ is the magnetic permeability of free space and *ε*_0_ is the electric permittivity of free space.

Since the strength of the induced electric field cannot be directly derived from the system of coupled ODEs in equation (3), the integral form of Ampere-Maxwell Law has to be used to describe the electromagnetic field generated by ionic and axial current passing during an action potential propagation.

$${\oint }_{C}\overrightarrow{B}\cdot \,\overrightarrow{d}\,l={\mu }_{0}({I}_{enc}+{\varepsilon }_{0}\frac{d}{dt}{\int }_{s}\overrightarrow{E}\cdot \hat{n}da)$$

As we have defined $\frac{d}{dt}{\int }_{s}\overrightarrow{E}\cdot \hat{n}da$ as the rate of change of electric flux, then the electric flux can be solved for the charge enclosed within the neuron to result in

$${\int }_{s}\overrightarrow{E}\cdot \hat{n}da={\int }_{s}\frac{\sigma }{{\varepsilon }_{0}}da=\frac{Q}{A{\varepsilon }_{0}}{\int }_{s}da=\frac{{Q}_{enc}}{{\varepsilon }_{0}}$$

Even though no charge is said to be contained within a conductor, which is what we are viewing the neuron in this case, since external voltage is constantly being applied onto the neuron during an action potential from the previous to the following segment, resulting in axial current propagation described in equations (8) and (9), then we cannot disregard the term in the Ampere-Maxwell equation that includes *Q*_*enc*_. This then results in spatio-temporal changes in the charges accumulated on the inside of the neuron. Since the action potential propagates with such a velocity to occur around *100 times in a second*, *Q*_*enc*_ is solely a function of charge accumulation due to ionic flow through sodium, potassium and other leak channels.

This then gives us the final version of the Ampere-Mawell equation that describes the strength of the electromagnetic field around a neuron. In order for it to be solved, the charge enclosed within the neuron needs to be precisely measured.

$${\oint }_{C}\overrightarrow{B}\cdot \,\overrightarrow{d}\,l={\mu }_{0}\overrightarrow{I}+\frac{{Q}_{enc}}{dt}$$

### Theoretical model for cell motion in the CNS

To account for cell motion within the electromagnetic field defined by the system of coupled ODEs, the cells are modeled as negatively-charged spheres that “behave like a Newtonian fluid with viscosity of 0.0012 Pa-s”^20^. As their flow is pressure driven and depends on the concentration gradient, it is best described using smoothed dissipative particle dynamics method during which they exhibit generalized Levy walks^21^, under the influence of the induced inhomogeneous electromagnetic fields around neurons that are proposed by this paper.

### Range of the magnetic field around the nodes of Ranvier and the myelinated axonal sections

In order to compute the range at which the magnetic field can act upon the cells, to see whether the magnetic fields acts upon large enough distances for cells to detect it, a simplified version of the model proposed in this paper was used, excluding the spatio-temporal propagation of the action potential and, therefore, excluding the spatial and temporal indexes from equations used. The reason this could have been done is because, even though the field itself is extremely inhomogeneous, at small enough distances around the neuron it can be seen as homogeneous and still following the Biot-Savart Law.

Treating the neuron as a wire with non-uniform current density, the line integral of the magnetic field strength can be solved as

$${\oint }_{C}\overrightarrow{B}\cdot \,\overrightarrow{d}l=2\pi r\overrightarrow{B}$$

Using the obtained expression in the Ampere-Maxwell equation,

$$2\pi r\overrightarrow{B}={\mu }_{0}I+\frac{{Q}_{enc}}{dt}$$

Since the field can be seen as homogeneous at relatively small distances, then the term including *Q*_*enc*_, the charge enclosed within the neuron, approaches 0 and can be disregarded. This results in

$$2\pi r\overrightarrow{B}={\mu }_{0}I$$

where I is the sum of the axial, longitudinal and ionic currents, as proposed by our model.

Disregarding the spatio-temporal variations of the currents and electromagnetic field, in order to obtain the distance at which the electromagnetic field acts the current flowing through the neuron is defined as

$$I={g}_{K}(V-{V}_{K})+{g}_{Na}(V-{V}_{Na})+{g}_{L}(V-{V}_{L})+\frac{V}{R}$$

where V is the membrane potential and R is the resistance of the neuron. For simplicity, *g*_*L*_(*V*−*V*_*L*_) = *0*.

Therefore,

$$I={g}_{K}(V-{V}_{K})+{g}_{Na}(V-{V}_{Na})+\frac{V}{\frac{\rho x}{2\pi r(x+r)}}$$

$$I={g}_{K}(V-{V}_{K})+{g}_{Na}(V-{V}_{Na})+\frac{2\pi r(x+r)V}{\rho x}$$

This yields an equation for the magnetic field of the form

$$\overrightarrow{B}(r)=\frac{{\mu }_{0}[{g}_{K}(V-{V}_{K})+{g}_{Na}(V-{V}_{Na})+\frac{2\pi r(x+r)V}{\rho x}]}{2\pi r}$$

#### Range of the magnetic field around the nodes of Ranvier

In order to obtain the homogeneity radius of the magnetic field, maximum values for all the constants from the Hodgkin-Huxley equation^22^ are taken to be:

$${V}_{r}-VNa=-\,115\,mV$$

$${V}_{r}-VK=12\,mV$$

$${g}_{Na}=28\,mS/c{m}^{2}$$

$${g}_{K}=12.5\,mS/c{m}^{2}$$

Next, since we are now working with the field around the node of Ranvier, according to Carcano *et al*.^23^ the length of the node of Ranvier is taken to be

$$x=1.08\pm 0.02\,\mu m,$$

and the density of the neuron is assumed to be *ρ* = *1 g/ml*, as there are no obtainable measured values.

In order to obtain the maximum distance from axolemma at which the magnetic field can act upon, $\overrightarrow{B}$*(r)* is set to 0 and the equation is solved for r.

$$\frac{{\mu }_{0}[{g}_{K}(V-{V}_{K})+{g}_{Na}(V-{V}_{Na})+\frac{2\pi r(x+r)V}{\rho x}]}{2\pi r}=0$$

$$\frac{1.2566\cdot {10}^{-3}[12.5\,mS/c{m}^{2}\cdot 12\,mV+28\,mS/c{m}^{2}\cdot (\,-\,115\,mV)+\frac{2\pi r(1.08\cdot {10}^{-4}cm+r)}{1\,g/ml}]}{2\pi r}=0$$

$$r={D}_{max}=6.606\,\mu m$$

#### Range of the magnetic field at the myelinated segments

For this computation, the steps were repeated as for the nodes of Ranvier but just the length of the myelinated segment, x, was set at

$$x=3.08\pm 0.02\,\mu m$$

Therefore, the distance on which the magnetic field acts upon, at the myelinated segments is

$$r=5.696\,\mu m,$$

but this distance includes the thickness of the myelin sheath, i.e. the full range of the magnetic from the surface of the neuron, axolemma. With this, the thickness of the myelin sheath has to be subtracted from this value to obtain the true distance, from the surface of the myelin sheath, neurilemma, whose thickness was taken to be an average of *d* = *3.63* ± *0.05* *μm*, at which the magnetic field is present.

With this, the real distance at which the magnetic field acts upon, starting from neurilemma, is

$${D}_{max}=2.066\,\mu m$$

### Modeling the maximum strength of the magnetic field around neurons

As it was shown, the neuron can be modeled as a current-carrying wire whose current strength varies according to the relative position of the node of Ranvier or the myelinated segment. If one such region is observed, the current flow at the node of Ranvier will induce a circular magnetic field at that region. The strength, direction and the force this magnetic field exerts on particles will then be fully determined by the total axial, longitudinal and ionic current flow within that segment.

Approximating the current within an axon to be steady in time over a small enough Δ*x* element of the node of Ranvier, the induced magnetic field can be said not to vary in time and be relatively homogeneous at distances of several microns around the axon, and Biot-Savart Law for a magnetic field around a wire can be used. If this was not the case and the current was varied in time even at such lesser scales, that would induce a time-varying magnetic field and a new source term called displacement current would have to be added, resulting in the Ampere-Maxwell equation; the one proposed in this paper to more faithfully describe the spatio-temporal propagation of the action potential and associated non-homogeneous electromagnetic fields.

According to Biot-Savart Law, the magnetic field around a conducting wire, which a neuron can be approximated as, is:

$$B=\frac{I{u}_{0}}{4\pi {R}^{3}}dL\times \hat{R}$$

Here, the goal is to compute the magnetic field, *B*, at position *r*, generated by a steady ionic, longitudinal and axial current, *I*, through a neuron. The electric current flow through the neuron is approximated as continual flow of ions through sodium, potassium and leak channels which is constant in time and results in no accumulation or depletion of charges at any point added to the longitudinal current flow resulting from impulse propagation.

**Figure 5:**
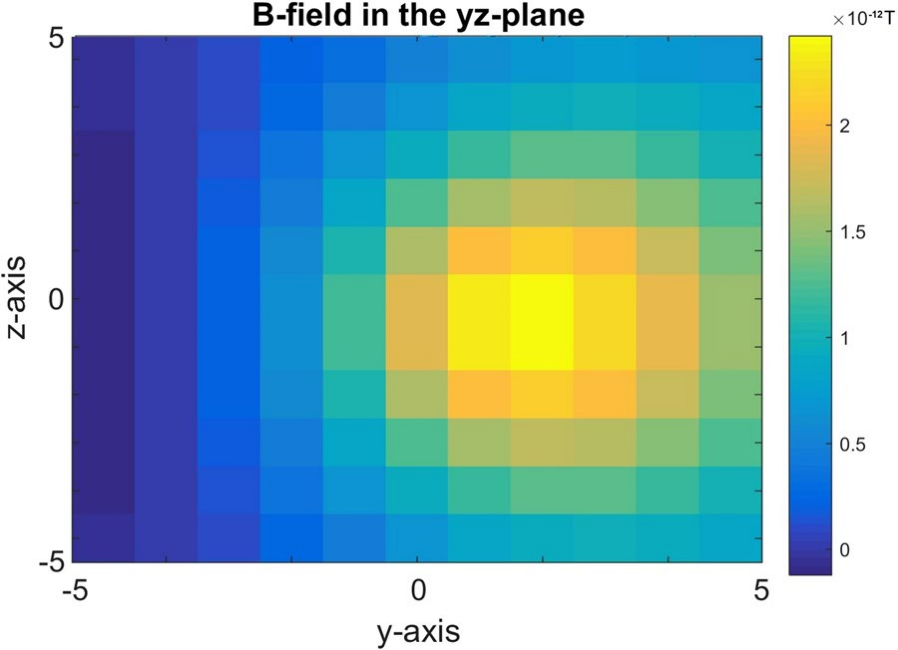
Color plot of magnetic field strength generated around the neuron in the y-z plane, with the legend indicating the strength of the magnetic field in T. Here, the magnetic field is seen to be the strongest, as expected, closest to the axon and non-linearly falls off with distance after reaching D_max_, displaying first signs of inhomogeneity. At its strongest, the magnetic field yields a value of *B* = *3.0* × 10^−12^ *T*.

For this, a source code was written in Matlab which consists of initialization of the x-, y- and z- components of the space in the form

$${X}_{w}=floor\frac{-N}{2}:floor\frac{N}{2}$$

$${Y}_{w}=zeros(N+1,1)$$

$${Z}_{w}=zeros(N+1,1)$$

and subsequent calculations of the vector components at each segment of the neuron. After defining x-, y- and z- components of the magnetic field around the neuron and situating it in the yz-plane, the code was looped through for each iteration to obtain the strength of the field in near proximity to the axon (Fig. 5).

As the magnetic field becomes increasingly inhomogeneous with an increase in the distance from the source, the Ampere-Maxwell equation has to be used past *D*_*max*_ in order to accurately depict the changes in the field strength that, ultimately, result in the magnetic field strength measured by MEG.

### *In-vivo* MRI cell tracking study following the impact that the cell surface charge modification has on its migration in the CNS

An *in-vivo* magnetic resonance imaging (MRI) study has been performed using C57Bl6 mouse in whom stroke has been induced by MCAO method, as reported by our group^24^. Upon isolation of T-lymphocytes from blood plasma obtained through orbital venous sinus bleeding, the cells were separated in two groups. The first group was treated with a glucocorticoid receptor antagonist RU486 (Mifepristone 98%, Sigma-Aldrich), which influences the net cell surface charge^5,25–27^, whilst the second one was not treated and served as a control. Both cell groups were labeled with a superparamagnetic iron oxide nanoparticles solution (SPION, Sigma Aldrich), enabling us to visualize them under an MRI.

After assuring that cells in both groups do exhibit the same rate of multiplication and viability, 4 mice with stroke were injected with cells treated with RU486 and other 4 with non-treated cells. One million of cells have been injected in the region of striatum (*AP* − 0.5, *ML* + 2.5 *and DV* − 2.5), approximately 500 *μm* from the outer border of the stroke. MRI Biospec Bruker 7 T has been used to detect the transplanted cells and to measure distances that the migrating cells reached, 7 days after the transplantation.

Although this experiment, in which we tested our hypothesis that surface charge neutralization will influence migration of T-lymphocytes, has been performed with several levels of control (i.e. we confirmed that there is no difference in cell viability prior to injection and have observed no single difference in cell physiology prior to injection), we plan to confirm our finding by additional experiments. The first one will be in the form of a *in-vivo* test with electromagnetic field sources and live cell imaging to track T-lymphocyte’s movement under the influence of external electromagnetic fields, be it in their charged or uncharged form. The second study will aim to measure the exact value of charge on T-lymphocytes and, subsequently, find the optimal charge distribution along its perimeter to maximize or minimize its migration – depending on the circumstances in question and the final desired effect.

## Data Availability

All data generated or analyzed during this study, except for datasets generated during the *in-vivo* MRI cell tracking study, are included in this published article. The data obtained by the *in-vivo* MRI cell tracking study are not publicly available due to the fact that work on those datasets is still being performed and will be published with the data obtained from the repeated and expanded experiments in process. Still, those datasets are available from the corresponding author on reasonable request.

## References

[1] Hoshiba, T., Yoshikawa, C. & Sakakibara, K. Characterization of initial cell adhesion on charged polymer substrates in serum-containing and serum-free media, *Langmuir, ACS Publications* (2018). 10.1021/acs.langmuir.8b00233 29544251

[2] Vogel H, Butcher EC, Picker LJ (1992). H-CAM expression in the human nervous system: evidence for a role in diverse glial interactions. J Neurocytol..

[3] Rajasekaran SA (2001). Na, K-ATPase *β*-subunit is required for fpithelial polarization, suppression of invasion, and cell Mmtility. Molecular Biology of the Cell..

[4] Li L (2017). Expression of the *β* 3 subunit of Na+/K+-ATPase is increased in gastric cancer and regulates gastric cancer cell progression and prognosis via the PI3/AKT pathway. Oncotarget..

[5] Mann CL, Cidlowski JA (2001). Glucocorticoids regulate plasma membrane potential during rat thymocyte apoptosis *in vivo* and *in vitro*. Endocrinology..

[6] Fan, X., Xie, J. & Tian, J. Reducing Cardiac Fibrosis: Na/K-ATPase Signaling Complex as a Novel Target. *Cardiovasc Pharm Open Access*. **204** (2017). 10.4172/2329-6607.1000204 PMC5638140 29034264

[7] Pielak RM (2017). Early T cell receptor signals globally modulate ligand:receptor affinities during antigen discrimination. Proceedings of the National Academy of Sciences of the United States of America..

[8] Dustin ML (1990). Role of adhesion molecules in activation signaling in T lymphocytes. Journal of Clinical Immunology..

[9] Gagnon E, Schubert DA, Gordo S, Chu HH, Wucherpfennig KW (2012). Local changes in lipid environment of TCR microclusters regulate membrane binding by the CD3*ε* cytoplasmic domain. Journal of Experimental Medicine..

[10] Yorulmaz S, Jackman JA, Hunziker W, Cho NJ (2016). Influence of membrane surface charge on adsorption of complement proteins onto supported lipid bilayers. Colloids Surf B Biointerfaces..

[11] Kyluik-Price DL, Scott MD (2016). Effects of methoxypoly (Ethylene glycol) mediated immunocamouflage on leukocyte surface marker detection, cell conjugation, activation and alloproliferation. Biomaterials..

[12] Kirschvink JL, Kobayashi-Kirschvink A, Woodford BJ (1992). Magnetite biomineralization in the human brain. Proceedings of the National Academy of Sciences of the United States of America..

[13] Catterall WA, Raman IM, Robinson HPC, Sejnowski TJ, Paulsen O (2012). The Hodgkin-Huxley heritage: from channels to circuits. The Journal of neuroscience:the official journal of the Society for Neuroscience..

[14] Hämäläinen M, Hari R, Ilmoniemi R, Knuutila J, Lounasmaa OV (1993). Magnetoencephalography–theory, instrumentation, and applications to noninvasive studies of signal processing in the human brain. Reviews of Modern Physics..

[15] Lukens JR (2015). NLRP12 negatively regulates autoinflammatory disease by modulating interleukin-4 production in T cells. Immunity..

[16] Nordenström, B. E. W. Exploring BCEC-systems, *Nordic Medical Publications* (1998).

[17] De Olyveira GM (2017). Surface physical chemistry properties in coated bacterial cellulose membranes with calcium phosphate. Mater Sci Eng C Mater Biol Appl..

[18] Shen, W. B. *et al*. Magnetic enhancement of stem cell–targeted delivery into the brain following MR-guided focused ultrasound for opening the blood–brain barrier. *Cell Transplantation*. 1235–1246 (2017). 10.1177/0963689717715824 PMC5657739 28933214

[19] Hodgkin, A. L. & Huxley, A. F. A quantitative description of membrane current and its application to conduction and excitation in nerve. *The Journal of Physiology*. 500–544 (1952). 10.1113/jphysiol.1952.sp004764 PMC1392413 12991237

[20] Furlani EP (2007). Magnetophoretic separation of blood cells at the microscale. Journal of Physics.

[21] Marth W, Aland S, Voigt A (2016). Margination of white blood cells: a computational approach by a hydrodynamic phase field model. Journal of Fluid Mechanics..

[22] Cole KS, Curtis HJ (1939). Electric impedance of the squid giant axon during activity. The Journal of General Physiology..

[23] Arancibia-Cárcamo, I. L. *et al*. Node of Ranvier length as a potential regulator of myelinated axon conduction speed. *eLife*. **6** (2017). 10.7554/eLife.23329 PMC5313058 28130923

[24] Kosi N, Alic I, Salamon I, Mitrecic D (2018). Stroke promotes survival of nearby transplanted neural stem cells by decreasing their activation of caspase 3 while not affecting their differentiation. Neurosci Lett..

[25] Jang JH (2013). RU486, a glucocortcoid receptor antagonist, induces apoptosis in U937 human lymphoma cells through reduction in mitochondrial membrane potential and activation of p38 MAPK. Oncol Rep..

[26] Brewer, J. A. *et al*. T-cell glucocorticoid receptor is required to suppress COX-2-mediated lethal immune activation. *Nature Medicine*. 1318–1322 (2003). 10.1038/nm895 12949501

[27] Winoto A, Littman DR (2002). Nuclear hormone receptors in T lymphocytes. Cell..

